# Protocol for scalable top-down fabrication of InP nanopillars using a self-assembled random mask technique

**DOI:** 10.1016/j.xpro.2023.102237

**Published:** 2023-04-19

**Authors:** Joshua Zheyan Soo, Parvathala Reddy Narangari, Chennupati Jagadish, Hark Hoe Tan, Siva Karuturi

**Affiliations:** 1 Department of Electronic Materials Engineering, Research School of Physics, The Australian National University, Canberra, ACT 2601, Australia; 2 School of Engineering, The Australian National University, Canberra, ACT 2601, Australia; 3 ARC Centre of Excellence for Transformative Meta-Optical Systems, Research School of Physics, The Australian National University, Canberra, ACT 2601, Australia

**Keywords:** Physics, Energy, Material sciences

## Abstract

Nanostructured III-V semiconductors are attractive for solar energy conversion applications owing to their excellent light harvesting and optoelectronic properties. Here, we present a protocol for scalable fabrication of III-V semiconductor nanopillars using a simple and cost-effective top-down approach, combining self-assembled random mask and plasma etching techniques. We describe the deposition of Au/SiO_2_ layers to prepare random etch mask. We then detail the fabrication of nanopillars and photocathodes. Finally, we demonstrate III-V semiconductor nanopillars as a photoelectrode for photoelectrochemical water splitting.

For complete details on the use and execution of this protocol, please refer to Narangari et al. (2021).^1^

## Highlights

• Simple and cost-effective approach for scalable top-down fabrication of InP nanopillars
• Protocol combines self-assembled random mask and plasma etching techniques
• Application of fabricated InP nanopillars in photoelectrochemical water splitting

## Abstract (editor highlights)

Publisher’s note: Undertaking any experimental protocol requires adherence to local institutional guidelines for laboratory safety and ethics.

## Graphical abstract

**Figure:**
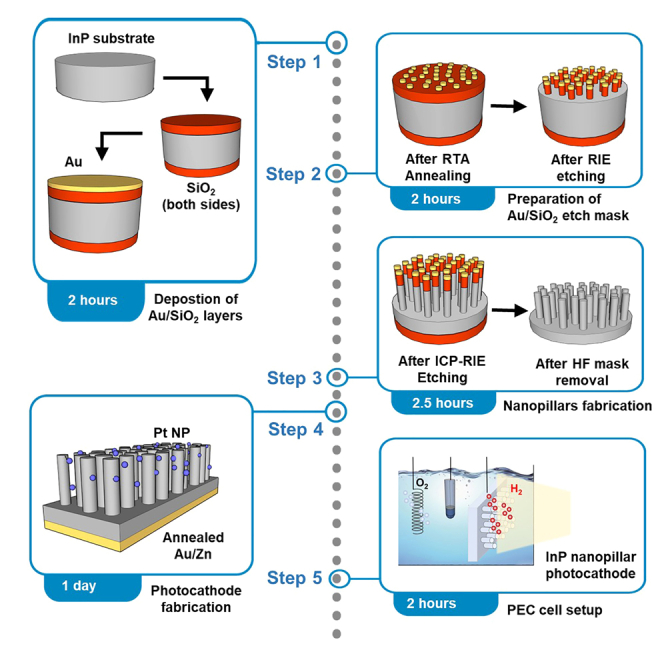

## Before you begin

Top-down fabrication method can be used to fabricate semiconductor nanopillars (NPs) with controlled dimensions, density with predefined doping, and heterostructures; which is challenging to achieve using bottom-up growth methods. The top-down method involves etching the material around the pre-patterned mask using either chemical or plasma etching. The anisotropic nature of plasma etching helps to achieve high aspect ratio NPs in the top-down approach. However, applying top-down fabrication of NPs is hindered due to the necessity of using complex, scalable, and expensive lithography masking techniques. Here, we developed a simple and scalable methodology, which constitutes self-assembled random mask technique and inductively coupled plasma reactive ion etching (ICP-RIE), for top-down fabrication of wafer-scale III-V semiconductor NPs. In this technique, a deposited thin metal film undergoes dewetting to form self-assembled random metal nano-dots when annealed at higher temperature. These randomly formed metal nano-dots are used as an etch mask for top-down fabrication of semiconductor NPs. The protocol below describes the specific steps involved in top-down fabrication of InP NPs using ICP-RIE and self-assembled random mask technique. This protocol consists of three sections (a) development of hard self-assembled Au/SiO_2_ random nano-mask using self-assembled random mask technique, (b) fabrication and plasma damage removal of InP NPs, and (c) fabrication and demonstration of InP NP photocathodes for photoelectrochemical (PEC) water splitting. Here, we used a commercial 350 μm, Vertical Gradient Freeze-grown, p-type InP wafer (zinc-doped, <100> orientation) for fabrication of InP NPs. However, we have also used this protocol for fabrication of other III-V semiconductor NPs such as GaN, InGaN/GaN and GaAs, as well as Si.

Further knowledge on top-down fabrication of semiconductor nanopillars can be found in the following literature.^1^^,^^2^

Please refer to the key resources table for the list of equipment needed for this protocol.

## Key resources table

**Table:** 

REAGENT or RESOURCE	SOURCE	IDENTIFIER
**Chemicals, peptides, and recombinant proteins**		

Monocrystalline InP wafer (p-type, 350 μm)	AXT Inc.	N/A
Sapphire wafer (101.6 mm diameter, 1.0 mm thickness)	Princeton Scientific	N/A
Hydrochloric acid (HCl, 36%)	Ajax Finechem	CAS: 7647-01-0
Hydrogen fluoride (HF, 48%)	Merck	CAS: 7664-39-3

**Other**		

High vacuum grease	Ajax Finechem	Model: AJA1400
Isopropanol	CHEM-SUPPLY Pty. Ltd.	CAS: 67-63-0
Cotton buds	Local vendor	N/A
Nail polish	Local vendor	N/A
Platinum coil counter electrode	BAS Inc.	Catalog Num: 012961
Ag/AgCl electrode	BAS Inc.	Catalog Num: 012167
Plasma enhanced chemical vapor deposition (PECVD)∗	Oxford Instruments	Product ID: Plasmalab 100 Dual Frequency
Electron beam (E-beam) evaporator∗	Ferrotec Temescal	Product ID: BJD-2000
Rapid thermal annealer (RTA)∗	Qualiflow	Product ID: JetFirst 100
Reactive ion etching (RIE)∗	Oxford Instruments	Product ID: Plasmalab 80
Inductively coupled plasma-reactive ion etching (ICP-RIE)∗	Samco	Product ID: RIE-400iP
Potentiostat∗∗	CH Instruments/Autolab	Product ID: CHI660E (CHI Instruments)/PGSTAT302N (Autolab)
Solar simulator (with 300 W Xe lamp and AM 1.5G filter)∗∗	Abet Technologies	SunLite™ Solar Simulator 100 watt (Product ID: 11002-2)
Ellipsometer ∗∗∗	JA Woollam	Product ID: M-2000D
UV-Vis spectrophotometer∗∗∗	PerkinElmer	Lambda 950
Time-resolved photoluminescence (TRPL) system∗∗∗	Custom setup	N/A
Scanning electron microscope (SEM)∗∗∗	FEI	Verios

***Note:*** ∗ for top-down InP fabrication; ∗∗ for PEC water splitting tests; ∗∗∗ for material characterization.

***Alternatives:*** In this protocol, we use an e-beam evaporator for the deposition of Au layer on the SiO_x_-layered InP wafer. A thermal evaporator or sputter deposition system can also be used as an alternative tool for this step. Separately, we use RIE for etching SiO_2_ to produce the SiO_2_/Au mask. ICP-RIE can also be used as an alternative tool for this step.

## Step-by-step method details

### Deposition of SiO_2_ and Au layers for random etch mask preparation

**Timing: 2 h**

An etch mask plays a critical role in the top-down fabrication of NPs, as the NP dimensions are defined by the pre-patterned etch mask. In this work, we developed Au/SiO_2_ hard random-aligned nano-dot etch mask using self-assembled random mask technique for ICP-RIE etching of InP NPs, shown in the subsequent major steps. Using SiO_2_ in addition to the Au metal increases the durability of etch mask in plasma etching and thereby helps to achieve high aspect ratio NPs. Development of Au/SiO_2_ random mask starts with plasma enhanced chemical vapor deposition (PECVD) of SiO_2_ followed by e-beam deposition of thin layer Au. Details of each step of depositing both SiO_2_ and Au films are presented in this section.

1. Deposition of SiO_2_ layer.
  ***Note:*** The presence of SiO_2_ layer in the etch mask is required to increase the endurance time of the etch mask in plasma to achieve higher aspect-ratio NPs. Moreover, having SiO_2_ deposited on both sides of the wafer helps to prevent possible Au contamination of InP and protect the wafer from thermal decomposition during the subsequent high temperature annealing process. SiO_2_ deposition here is carried out using PECVD on both sides of the InP wafers.
  a. Blow-dry a clean p-InP wafer with nitrogen gun.
  b. Load p-InP wafer onto the metallic wafer carrier in the PECVD load-lock chamber and transfer it into the deposition chamber.
  c. Deposit 400 nm SiO_2_ layer under 81 sccm of N_2_, 355 sccm of N_2_O and 4.8 sccm of SiH_4_ at 300°C at 20 W RF power and 650 mTorr chamber pressure.
    ***Note:*** Chamber preconditioning is highly recommended to avoid cross contamination of deposited films. For this, a dummy deposition run is carried out before actual deposition onto the p-InP wafer. It is also important to calibrate the deposition rate of SiO_2_ using ellipsometry to deposit the SiO_2_ of required thickness.
  d. Allow time for cooling of the p-InP wafer after SiO_2_ layer deposition before collecting from the chamber.
    **CRITICAL:** InP wafers and the wafer carrier remains at high temperature immediately after coming out from the deposition chamber. Therefore, touching wafers and wafer carrier immediately after transfer from chamber to load-lock can cause skin burns. Also, handling the wafers with plastic tweezers can result in their softening. Therefore, it is advisable to wait for a few minutes before unloading the wafers from the load-lock chamber using metal tweezers.

2. Deposition of a thin Au metal film.
  ***Note:*** E-beam evaporation system is employed for the deposition of a thin Au film on InP wafers pre-deposited with SiO_2_. Thin metal film plays an important role in fabrication of NPs as their dimensions rely on the metal mask resistance in the plasma etching environment. Au is the metal of choice here as it can undergo dewetting at a temperature below the onset of InP decomposition (<450°C).
  a. Blow dry the SiO_2_ coated p-InP wafer using nitrogen gun.
  b. Load wafer into electron beam evaporator chamber.
    **CRITICAL:** The p-InP wafer should be immediately loaded to the electron beam evaporator chamber after SiO_2_ layer deposition and measurement to minimize surface contamination from ambient dust particles.
  c. Set the pressure to 9 × 10^-6^ Torr and pump the chamber.
  d. Once the vacuum reaches 9 × 10^-6^ Torr, the evaporation of 10 nm Au layer is carried out at 0.5 Å/s.
    **CRITICAL:** It is important to monitor the Au film thickness during this step, as a small change in film thickness can significantly change the dimensions and the density of Au nanodots and subsequently the NPs. For example, an increase in Au film thickness is expected to decrease the density of the NPs decrease and increase the diameter of the NPs. Excess Au thickness may also hinder effective de-wetting process which results in irregularly shaped random mask.
  e. Set the rotation speed of the wafer holder to 50 rpm to achieve the uniform deposition across wafer.
  f. Allow time for cooling of evaporation boat and evaporation system before collecting Au deposited SiO_2_/p-InP wafer from the chamber.

### Preparation of self-assembled Au/SiO_2_ random etch mask

**Timing: 2 h**

Following the deposition of SiO_2_ and Au films on InP wafer, the samples are then annealed at high temperature in RTA system to convert the thin Au layer into random Au nano-dots through a de-wetting process. In the next step, reactive ion etcher (RIE) is used to etch SiO_2_ around the Au nano-dots. Details of each processing step involved in developing Au/SiO_2_ random etch mask are presented in this section.

**Figure 1:**
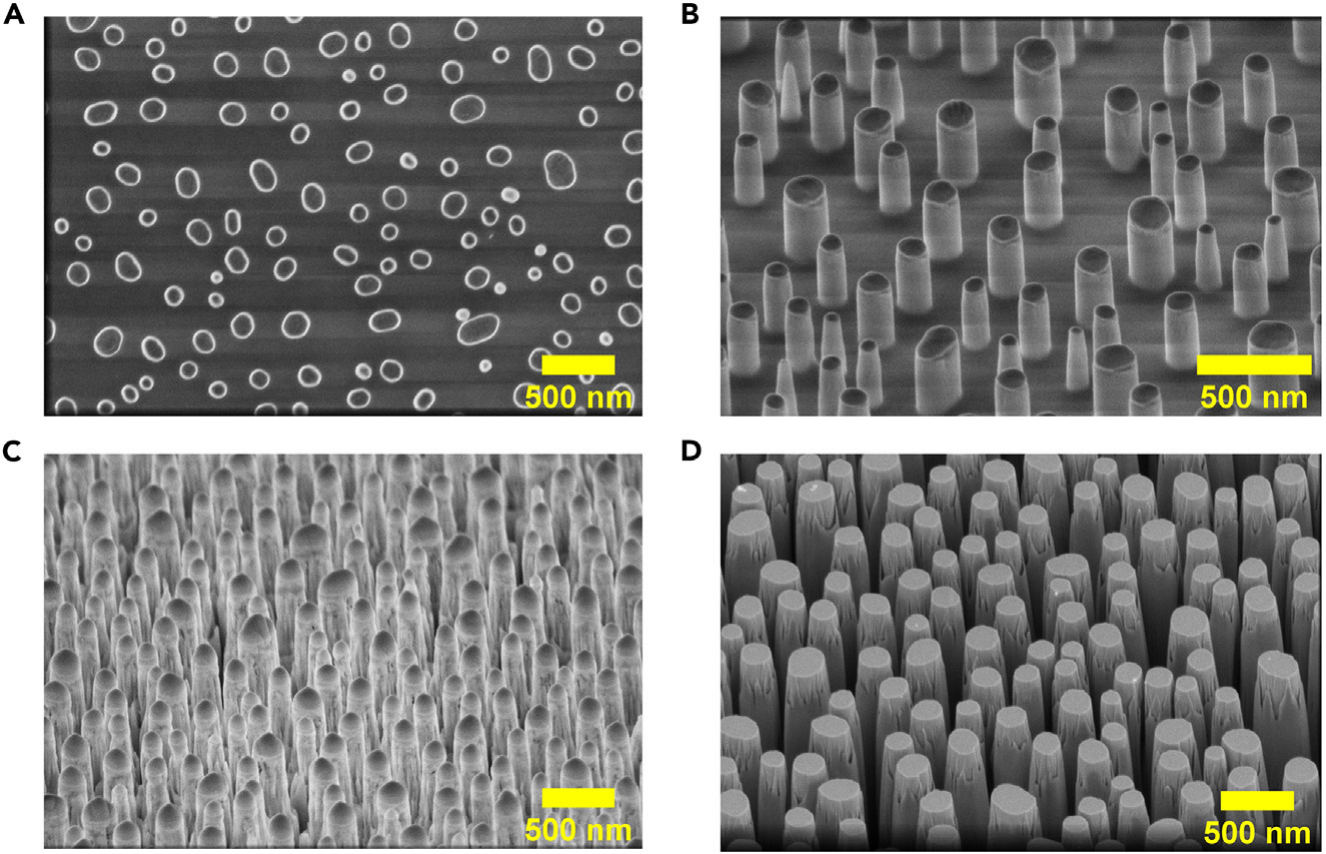
SEM images of various stages of top-down InP NPs fabrication (A) Au metal de-wetting after RTA annealing. (B) After RIE of the exposed SiO_2_ region/layer. (C) After ICP-RIE of InP. (D) After HF removal of the Au and SiO_2_ mask.

3. Producing self-assembled random Au nano-dot metal mask.
  ***Note:*** To produce the Au nano-dots metal mask, the Au deposited SiO_2_/p-InP wafer is rapidly annealed to induce the metal de-wetting process to form randomly aligned Au nano-dots. This step should be carried out shortly after Au layer deposition to avoid contamination of the Au film from ambient environment, which makes it difficult to convert metal film into randomly oriented nano-dots.
  a. Blow dry the wafer.
  b. Immediately transfer the wafer on to the RTA graphite sample holder.
    **CRITICAL:** The wafer should be immediately loaded to the RTA sample holder to minimize surface contamination from ambient dust particles.
  c. Anneal the InP wafer at 450°C for 9 min under 500 sccm of Ar and N_2_ gas.
    **CRITICAL:** Annealing at high temperature (beyond 500°C) can cause the destabilization and decomposition of the InP wafers which can release hazardous substances. While SiO_2_ is deposited on both side of the wafers to avoid wafer decomposition, ensure that there are no pin holes in the SiO_2_ layer. Use a tightly sealed annealing chamber with proper ventilation for suction of hazardous substances which may arise in adverse scenarios.
    ***Note:*** It is highly recommended to carry out an annealing trial/dummy run without any sample loaded before loading the InP wafer onto the sample holder.
  d. Allow time for cooling of InP wafer before collecting from the sample holder (see Figure 1A for outcome).

4. Fabrication of self-assembled Au/SiO_2_ random mask.
  ***Note:*** Fabrication of self-assembled random Au/SiO_2_ mask involves RIE of SiO_2_ around the Au nano-dots. It is important to run the calibration etching on bare SiO_2_ film to find out the etch rate of SiO_2_.
  a. Carry out plasma chamber cleaning for 10 min using Ar/O_2_ plasma at room temperature (approximately 22°C–25°C) under 100 mTorr. Plasma was created by flowing Ar/O_2_ gases (100 sccm) with applying RF power of 200 W.
  b. Blow dry the self-random nano-dots masked InP wafer with nitrogen gun.
  c. Load wafer into RIE etching chamber.
  d. Pre-condition the chamber using the same etching conditions used for SiO_2_ etching prior to running plasma etching on Au nano-dots masked samples.
  e. Once vacuum of 30 mTorr is reached, carry out SiO_2_ layer using CHF_3_ plasma at room temperature (approximately 22°C–25°C) for 13 min and 30 s. The CHF_3_ plasma was created by applying RF power of 200 W and maintaining continuous flow of 25 sccm CHF_3_ into plasma chamber.
    **CRITICAL:** It is important to know the etch rate of SiO_2_ film before RIE etching of real sample to avoid either over etching or under etching of SiO_2_ layer. To calibrate the etch rate, carry out etching on bare SiO_2_ film for 5 min. Measure the thickness of SiO_2_ film before and after plasma etching using ellipsometry. The difference in the thickness of SiO_2_ before and after the RIE etching divided by the etch time gives the etching rate of SiO_2_ under that particular RIE plasma condition.
  f. Retrieve the wafer from the etching chamber.
  g. Employ the scanning electron microscope to study the morphology and dimensions of self-assembled Au/SiO_2_ random mask.

### Fabrication and plasma damage removal of InP NPs

**Timing: 2.5 h**

This section presents the ICP-RIE etching of self-assembled Au/SiO_2_ random masked InP wafers for fabrication of InP NPs. Following that, it provides the procedure to remove the remnants of the Au/SiO_2_ mask on the InP wafer. As-fabricated NPs also suffer from surface defects caused by the impingement of high energy plasma ions at the surface of NPs during etching. These surface defects are detrimental for device performance as they act as undesired recombination centers for photogenerated charge carriers. Diluted HCl is employed to remove the surface plasma damage after the mask removal.

5. Fabrication of InP NPs using ICP-RIE etching.
  ***Note:*** This step covers the fabrication of the InP NPs after the formation of Au/SiO_2_ etch mask. This step is carried out by an anisotropic etching of the InP around random Au/SiO_2_ nano-dots using ICP-RIE.
  a. Set the ICP-RIE plasma chamber temperature at 200°C.
  b. Carry out ICP-RIE chamber cleaning for 25 min using Ar/O_2_ plasma set at 10/50 sccm using RF/ICP power of 50/300 W. The chamber pressure is maintained at 5 Pa.
  c. Clean a Ni-coated sapphire carrier wafer by wiping it using lint-free cloth dipped with isopropanol followed by immediate drying with nitrogen gun.
  d. Blow dry the Au/SiO_2_ random-masked InP wafer.
  e. Apply a small amount of thermal sink paste using cotton buds on the center of the rear side of the wafer.
    ***Note:*** Plasma etching of samples can result in rise of the sample temperature which influences the uniformity of the NPs height and morphology. Applying thermal sink paste helps to maintain the temperature of wafer. However, excessive use of thermal sink paste can reduce the ohmic conductivity of the rear contacts and influence the device performance made from these NPs.
  f. Place the wafer in the middle of the carrier wafer and load it into the etching chamber.
  g. Plasma etch the InP wafer with the Au/SiO_2_ mask at 200°C using SiCl_4_/Ar plasma at a flow rate of 1.5/25 sccm and RF/ICP power of 50/200 W. The chamber pressure is maintained at 0.15 Pa.
  h. Allow time for cooling of InP wafer and sapphire wafer before retrieving it from chamber (see Figure 1C for outcome).
  i. Remove InP wafer from the sapphire wafer and apply some isopropanol on the rear side of the wafer using cotton buds to remove the thermal sink paste.
6. Reminiscent Au/SiO_2_ mask removal.
  a. Prepare an etch solution for the mask layer removal by mixing 10 mL of HF (48%) with 40 mL of DI water in a Teflon beaker.
    ***Note:*** Careful attention to safety is required when conducting processes related to the use of HF acid solution. HF easily penetrates the skin and causes destruction of the deep tissue layers and bone. Personal protective equipment such as plastic face shield, chemical-resistant apron and neoprene rubber gloves must be worn while handling and using HF solution of any concentration.
    ***Note:*** Teflon beakers must be used in preparing HF solution to avoid corrosion of glass beakers.
  b. Immerse the sample in the diluted HF solution for 4 min to remove the Au/SiO_2_ mask at the top of NPs and SiO_2_ layer at the rear of wafer.
  c. Take out the sample from the diluted HF solution and wash it thoroughly by immersing in DI water (see Figure 1D).
  d. Blow dry the InP nanopillar sample with nitrogen gun.
  e. Study the morphology of NPs using SEM.
7. Removing plasma damage to NPs.
  ***Note:*** As-fabricated NPs suffer from defects caused by the impingement of high energy plasma ions at the NPs surface during ICP-RIE etching. These defects are detrimental for device performance as they act as undesired recombination centers for photogenerated charge carriers. Diluted HCl is employed to remove the plasma damage that occurred to the NPs surface after removing the mask remnants.
  a. Prepare a diluted HCl solution by adding 10 mL of HCl (36%) to 60 mL of DI water.
  b. Immerse the InP NP wafer into the diluted HCl solution for 15 min to remove the damaged surface layer.
    ***Note:*** Care should be taken to observe the variations in color of the InP wafer during HCl immersion in this step. A change in color from black to lighter grey indicates permanent damage and loss of the NPs due to excess HCl etching.
  c. Retrieve the InP NP wafer and wash it with DI water.
  d. Blow dry the InP NP wafer with nitrogen gun.
  e. Measure and compare the minority carrier lifetime of the NPs and bare InP wafer using TRPL to confirm the removal of plasma damage.

### Fabrication and PEC measurement of NP photocathodes

**Timing: 1 day**

**Figure 2:**
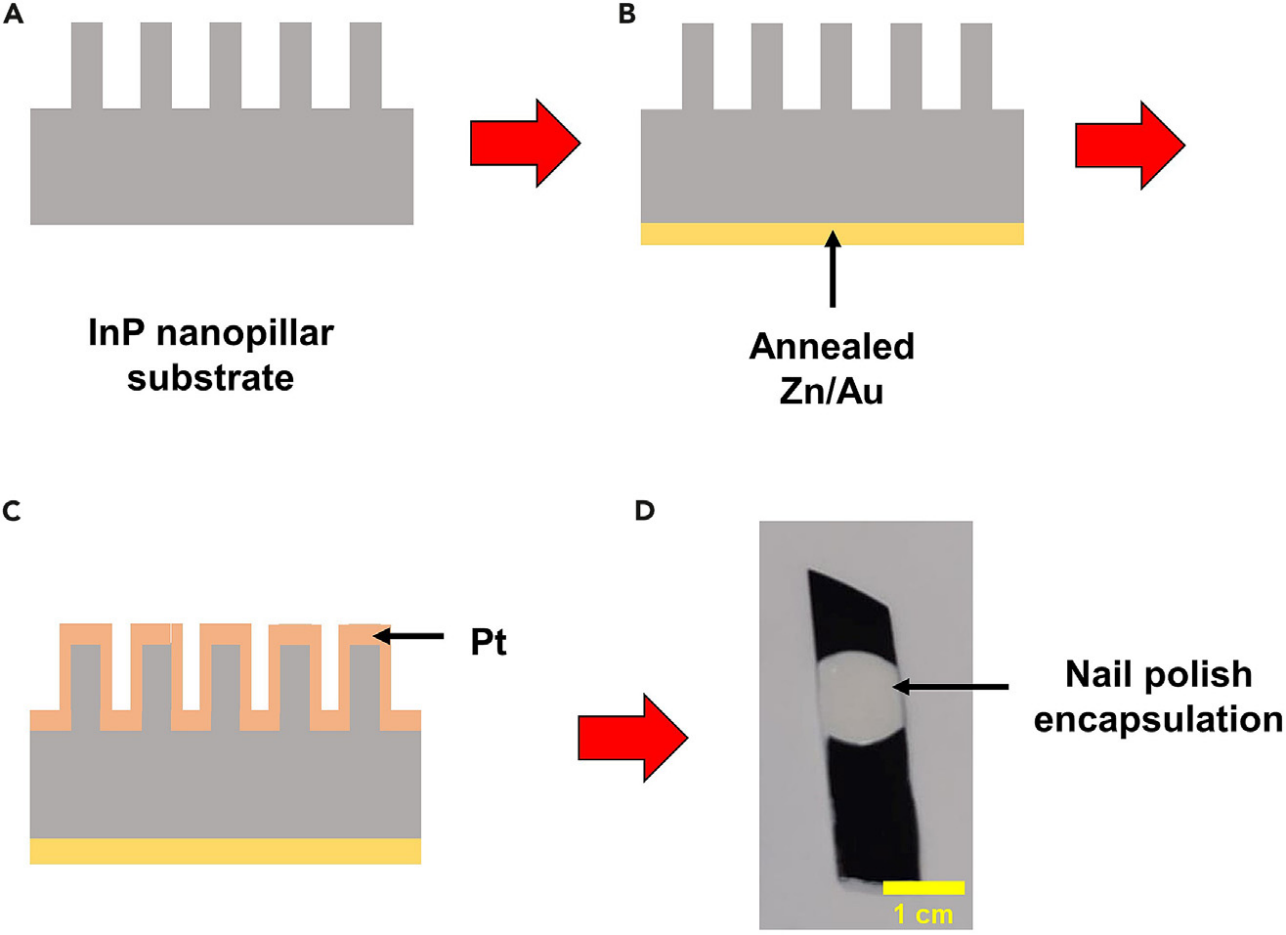
Key fabrication steps of InP NPs photocathode (A–C) Illustration of layer deposition on the rear and front of InP NPs wafer. (D) Photograph of final fabricated photocathode electrode.

This section of protocol describes the process steps involved in utilizing the InP NPs for fabrication of NP photocathode devices and measurement of their PEC performance. Sputter deposition and e-beam evaporation tools are employed for deposition of ohmic contacts and catalysts, respectively to finish the photocathode device fabrication. Figure 2 illustrates the key device fabrication steps for the photocathode device.

**Figure 3:**
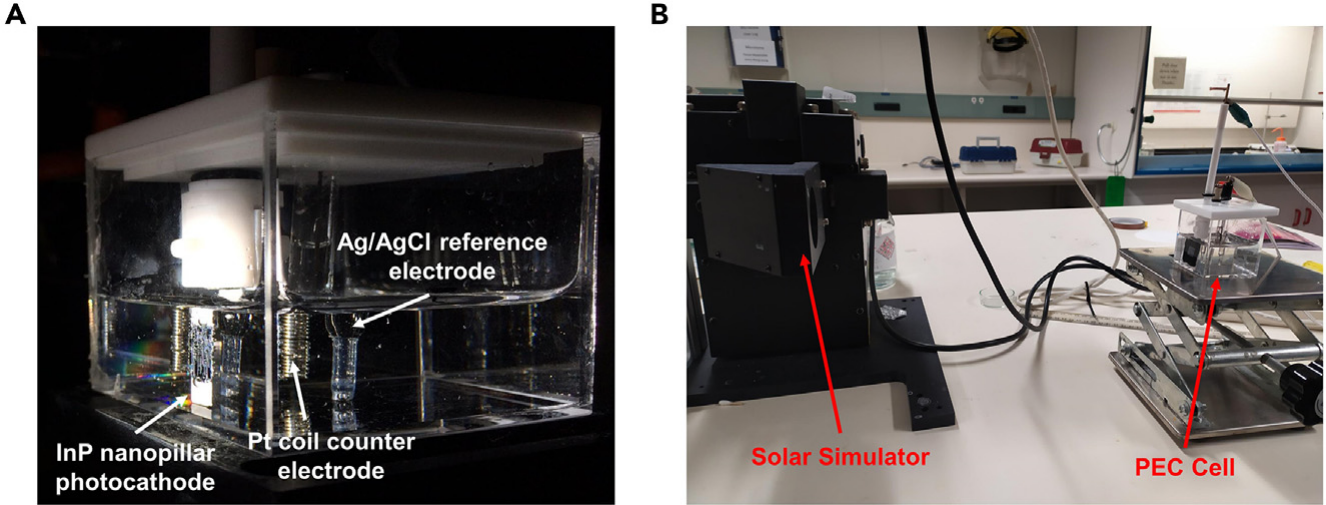
Setup of PEC water splitting measuring system (A) Photograph of electrodes immersed in 1.0 M KOH electrolyte filled quartz cell. (B) Photograph of measurement system with the solar simulator lamp source.
  d. Illuminate the flat region of the quartz cell with the solar simulator and calibrate to 1 sun intensity by adjusting the distance from the lamp source to the quartz cell surface (see Figure 3B).
  e. Perform a cyclic voltammetry sweep for 5 cycles followed by linear sweep voltammetry measurement in the anodic direction at scan rate of 50 mV/s.
  f. After data collection, retrieve the sample and wash with deionized water before drying with nitrogen gun.

8. Ohmic contacts for InP photocathode.
  ***Note:*** DC/RF sputter deposition is employed for the deposition of Zn/Au ohmic contacts at the rear of InP NPs wafer. After sputter depositions, samples are annealed in furnace to achieve low contact resistance contacts.
  a. Place the rear side of the InP NP sample (Figure 2A) facing upwards on the sputter holder and secure it using Kapton tape.
  b. Transfer to the sputter deposition chamber.
  c. Once vacuum of 4 × 10^-5^ mbar is reached, sputter 20 nm of Zn followed by 100 nm of Au using DC power supply at room temperature (approximately 22°C–25°C). Ar gas flow is set at 20 sccm while chamber pressure is set at 2.5 mTorr.
  d. Retrieve wafer from the loading chamber.
  e. Pre-heat the tube furnace to a temperature of 400°C.
  f. Place the sample in quartz boat and transfer it into the furnace using a quartz rod.
  g. Anneal the wafer in furnace at 400°C for 40 min under 20 sccm of N_2_/H_2_ (95%/5%) gas flow (Figure 2B).
  h. Allow the wafer and quartz boat to cool down before retrieving from tube furnace.
    **Caution**: Furnace surfaces could be hot. Proper protection and precautions should be taken while using the furnace.

9. Pt catalyst deposition and device preparation of InP photocathode.
  a. Blow dry sample with nitrogen gun.
  b. Immediately transfer to the e-beam evaporator.
  c. Once vacuum of 9 × 10^-6^ Torr is reached, deposit 4 nm of Pt at deposition rate of 0.5 Å/s on top of the sample under a constant rotation of 50 rpm (Figure 2C).
  d. Retrieve sample and blow dry with nitrogen gun.
  e. After finishing Pt catalyst deposition, paint the on the front side photocathode with nail polish to define the active area of photocathode.
  f. Rest them for 2 h to dry the nail polish.
  g. After nail polish on the front side has dried, apply nail polish at the rear of the sample to protect ohmic contacts from corrosion and avoid the carrier leakage into the electrolyte. Leave a small area of the metal contact to connect it to the holder for PEC measurements.
  h. Leave the fabricated NPs photocathode sample in ambient atmosphere to dry for 18 h at room temperature (approximately 22°C–25°C) (Figure 2D).
10. PEC water splitting performance measurement.
  ***Note:*** This section of the protocol describes the measurement of the PEC water splitting performance of the Pt-coated InP NPs photocathode. A 3-electrodes cell configuration is used in this setup. A Pt coil and Ag/AgCl (3.0 M NaCl) are used as counter and reference electrodes, respectively. A solar simulator is used as the light source, with the light intensity set to 1 sun using a reference Si solar cell.
  a. Prepare a 250 mL 1.0 M HCl solution by first diluting 21.0 mL of 36% HCl solution in 62.5 mL deionized water using magnetic stirrer. Top up the remainder of the volume required with deionized water until 250 mL final solution is reached.
  b. Pour 1.0 M HCl solution into a quartz cell.
  c. Set up a 3-electrode configuration with the InP photocathode, Pt coil and Ag/AgCl electrode as working, counter and reference electrodes, respectively (see Figure 3A).

## Expected outcomes

**Figure 4:**
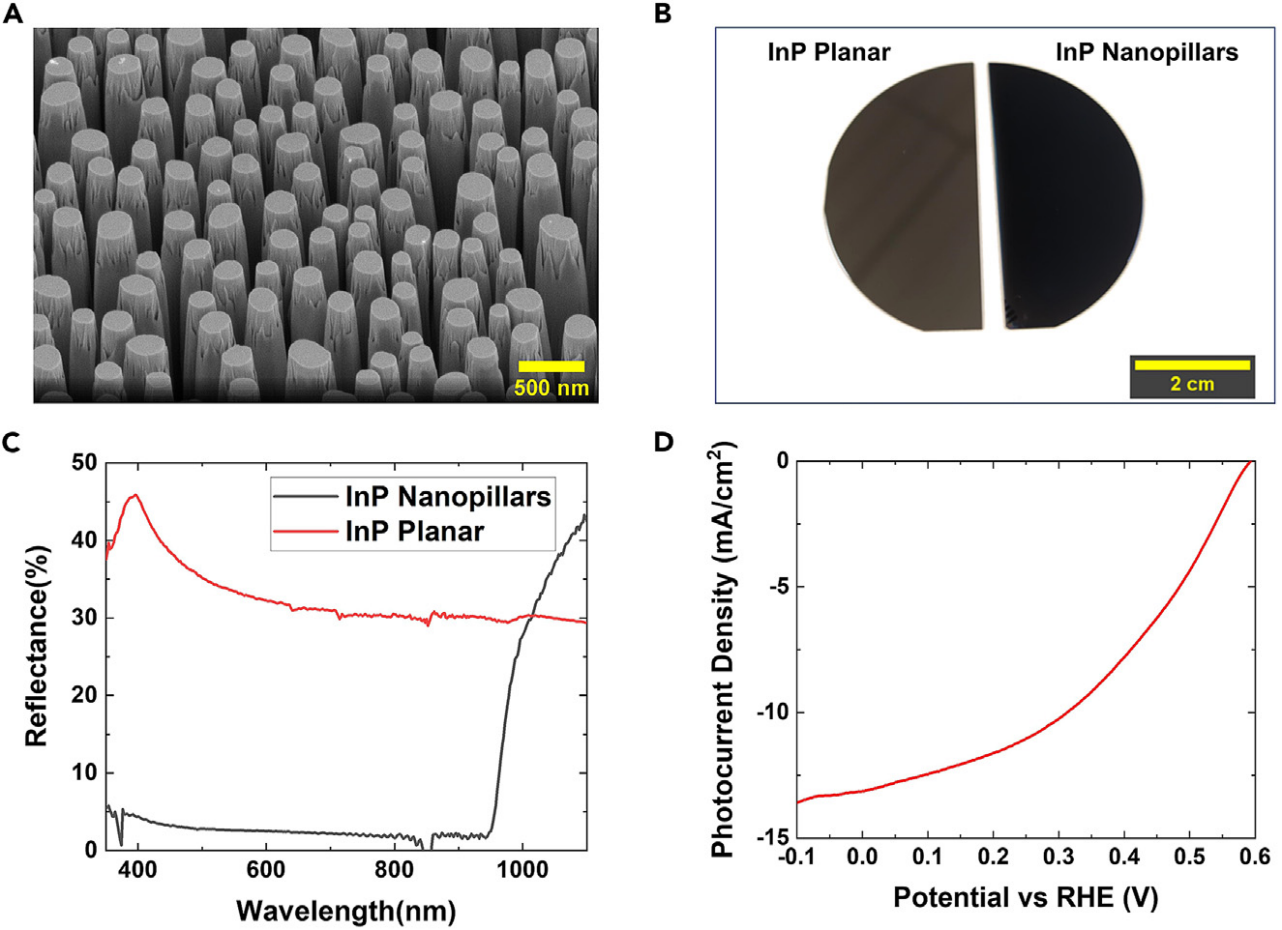
Expected outcomes and comparative performance of InP (planar and NPs) (A) SEM image of the InP NPs. (B) Photograph of side-by-side comparison of InP wafer and NPs. (C) UV-Vis reflectance of InP wafer and NPs. (D) Cathodic LSV sweep of InP NPs photocathode in both light and dark conditions.

This protocol provides the procedure for the simple, scalable top-down methodology for fabrication of InP NPs, using ICP-RIE assisted by a self-assembled randomly aligned Au/SiO_2_ mask. Fabrication of InP NPs starts with deposition of SiO_2_ followed by Au deposition. After the Au layer deposition and annealing, nanodot structures are formed throughout the wafer. Subsequently, SiO_x_ and InP are conformally etched along the dimensions of the Au nanodots to form InP nanopillars with controlled thickness. NPs of about 1.5 μm height are obtained using this fabrication method and conditions (Figure 4A).

InP NPs also have a noticeably non-reflective surface compared to the shiny surface of planar InP (Figure 4B). This is due to excellent absorption properties of NPs compared to their planar counterpart InP, particularly in the 400–1,000 nm wavelength (Figure 4C).

With respect to PEC water splitting, a conformal layer of TiO_2_ and Pt cocatalyst are deposited on the InP NPs. The resulting photocathode device exhibits an onset potential and saturated photocurrent density of approximately 0.6 V and 13 mA/cm^2^, respectively (Figure 4D).

## Limitations

This protocol allows fabrication of randomly positioned InP NPs via top-down etching method. However, it is difficult to realize precise control of the intervals between NPs and the diameter of the NPs themselves due to the random de-wetting nature of the Au film during mask formation. Other masking techniques such as e-beam lithography can be used to ensure a more precise control of the nanopillar diameter.^3^ This top-down methodology suits applications where improved light absorption properties are crucial.

This protocol also requires using a suite of fabrication equipment (e.g., PECVD, e-beam evaporator, RTA, ICP-RIE) to accomplish it. Therefore, there is a degree of complexity in optimizing the various parameters in each equipment to achieve the desired outcomes in each step of this protocol. However, optimizing the top-down etching parameters are relatively simpler compared to the more complex epitaxial bottom-up methods (e.g., MOCVD) typically employed for nanostructure fabrications.

InP substrates are also expensive to use for photoelectrode device fabrication, as is typically the case for III-V semiconductors. This can be remedied by using cheaper semiconductors such as Si as the substrate material of choice. It is worth noting that the same fabrication parameters cannot be used here, and optimization is required for each step to achieve similar results. Another cost-effective option is to use thin layer III-V semiconductors that are fabricated using epitaxial lift-off and/or spalling techniques before nanostructuring fabrication is carried out on them.

**Figure 5:**
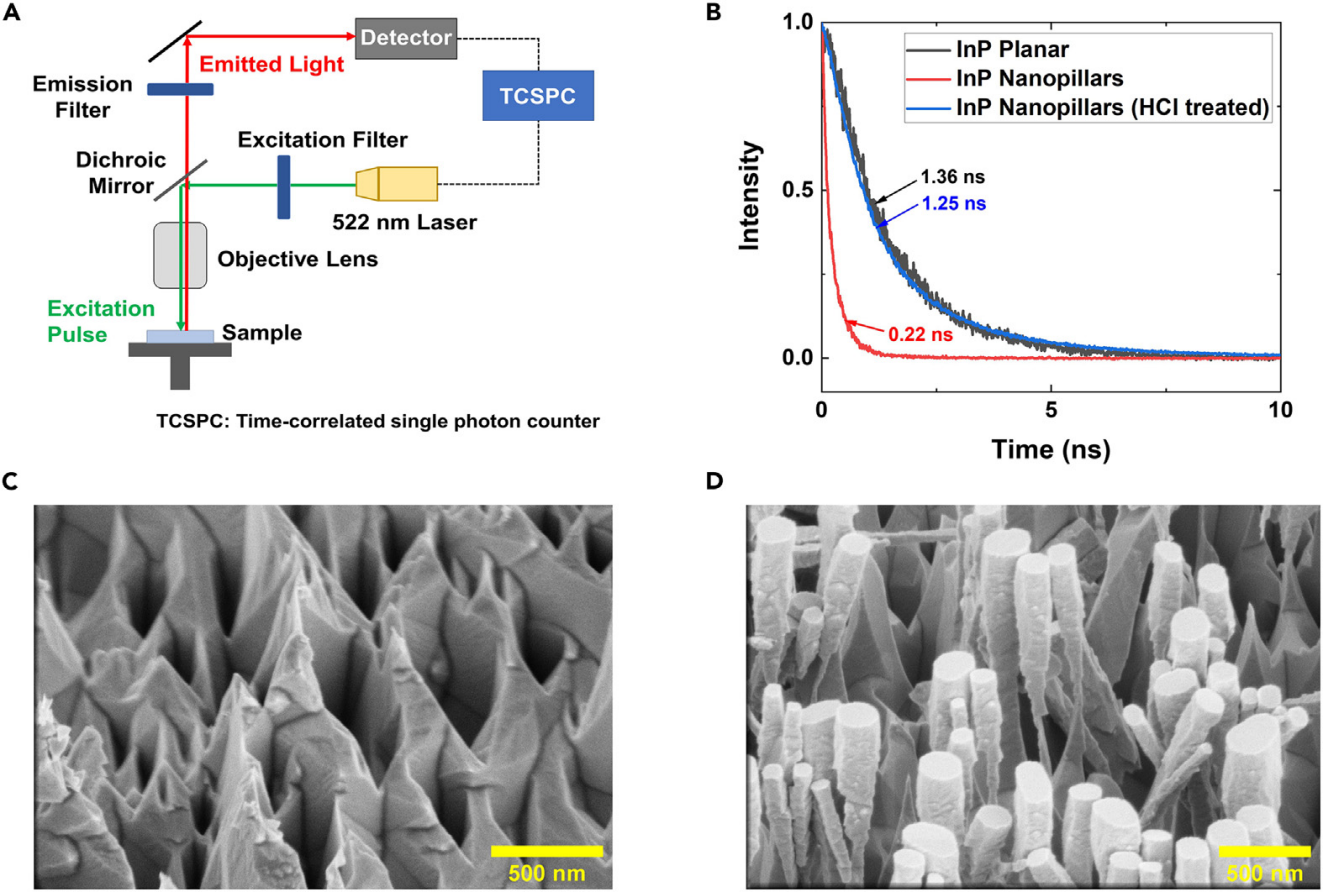
Carrier lifetime optimization of InP nanopillar wafers via TRPL (A) Schematic of the TRPL setup. (B) TRPL decay curves of planar InP and both non and HCl-treated InP nanopillars (carrier lifetime values included). (C and D) SEM images of over-etched InP NPs in diluted HCl solutions.

As-fabricated InP NP photocathodes may show low photocurrent density due to the low carrier lifetime of the InP NPs. This is a result of presence of surface defects on the InP NPs sidewalls as an undesirable effect of the ICP-RIE etching. The surface defects promote charge carrier recombination, which reduces its participation in water splitting reactions. The prescribed surface cleaning conditions (step 7, ‘fabrication and plasma damage removal of InP NPs’) may not entirely remove all surface damages present, as it is highly dependent on the amount of surface damage present and the wet-etching rate by diluted HCl. It is also why this is the most difficult step to accomplish in the entire fabrication process of the InP NPs, due to the inconsistent and highly sensitive nature of the wet-chemical plasma damage removal. As such, it is highly recommended that an individual optimization process of the charge carrier lifetime is carried out before device fabrication, whereby the carrier lifetime of the cleaned InP NPs is measured and optimized using a TRPL system (Figure 5A). Ideally, the measured carrier lifetime of the cleaned InP NPs should be approximately close to planar InP (Figure 5B). However, care is required during the optimization process to ensure that the NPs are not over-etched and structural integrity is uncompromised (Figures 5C and 5D). It is therefore crucial to maintain precise control of the concentration of the diluted HCl solution and the etching time once optimal etch parameters are attained. Meanwhile, it is also recommended to measure the carrier lifetime at different points of the sample to ensure plasma damage removal is consistent throughout the sample.

## Troubleshooting

### Problem 1

Highly irregular and non-uniform shape of Au mask after RTA annealing (step 3 in ‘preparation of self-assembled Au/SiO2 random etch mask’)”.

### Potential solution

**Figure 6:**
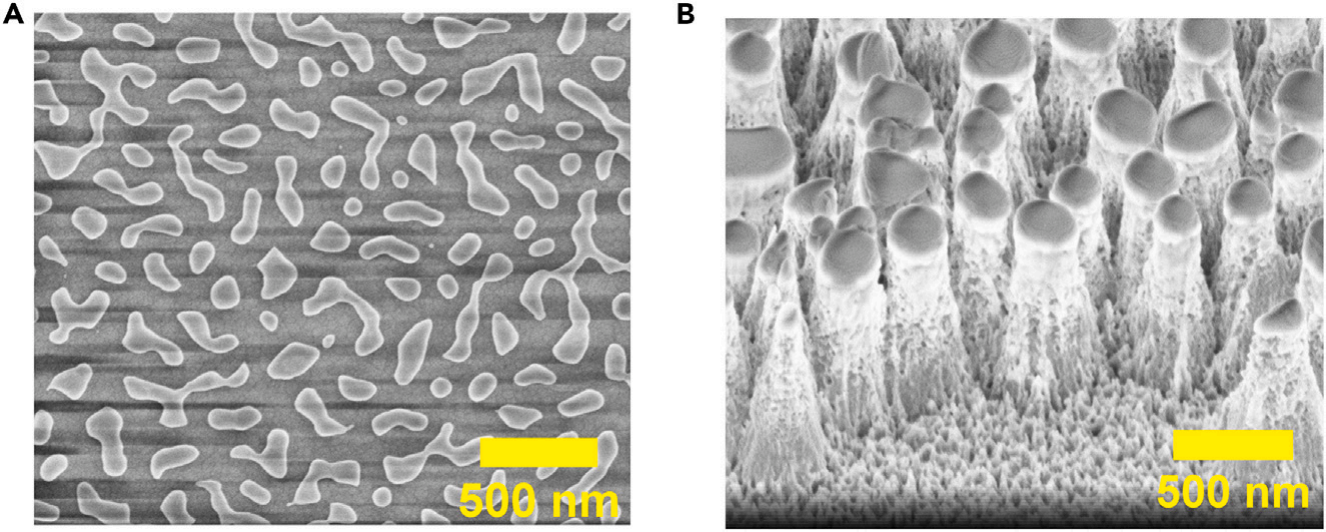
SEM images of problems encountered in InP NPs fabrication (A) Incomplete de-wetting of the Au film after RTA annealing. (B) Micromasking effects on InP NPs surface after ICP etching.

The non-uniformity of the Au mask shape is due to incomplete de-wetting of the Au layer during RTA annealing (Figure 6). The incomplete dewetting might be associated with high Au layer thickness, excess surface roughness of the underlying SiO_x_ layer or from impurities deposited during PECVD deposition. The Au layer thickness can be reduced during e-beam evaporation to ensure adequate thermal permeation across the film to induce complete de-wetting.^4^ Alternatively, the deposition rate of SiO_x_ can be lowered during PECVD by tuning down the precursor gas flows (N_2_, N_2_O, SiH_4_), to allow for a smoother SiO_x_ film. It is also recommended to carry out periodic cleaning of the PECVD chamber to avoid unwanted contamination of the SiO_x_ film. Figure 1A shows the correct outcome of the fully-dewetted Au mask if this solution is applied.

### Problem 2

Micromasking during ICP-RIE process leading to rough surfaces on nanopillars sidewall after etching (step 5 in ‘fabrication and plasma damage removal of InP NPs’).

### Potential solution

Possible contamination from high concentration of InCl_3_ in the chamber as a by-product from previous etching processes may lead to their adsorption onto the InP wafer which could further result in micromasking of InP surface^5^ (Figure 6B). This can be remedied by performing a longer chamber cleaning process using O_2_/Ar plasma after several etching cycles. InP NPs with smoother sidewalls and base can be obtained if this solution is applied (Figure 1C).

## Resource availability

### Lead contact

Further information and requests for resources and reagents should be directed to and will be fulfilled by the lead contact [Siva Karuturi] (siva.karuturi@anu.edu.au).

### Materials availability

This study did not generate new unique reagents.

### Data and code availability

Data would be made available upon request.
